
JOURNAL INFORMATION
==============================
NLM Title Abbreviation: Innov Surg Sci
Journal ID: Innov Surg Sci
NLM Journal ID: 101708165
Journal ID: iss

Innovative Surgical Sciences

EISSN: 2364-7485
Publisher: De Gruyter

ARTICLE INFORMATION
==============================
PMCID: PMC11005949
DOI: 10.1515/iss-2024-2004
Article ID: iss-2024-2004
Article version: 1
Subjects: Article

Abstracts DGG

Electronic publication date: 2024 Apr 9
Publication date: 2024 Apr
Volume: 9
Issue: Suppl1
First page: 204
Last page: 208
Copyright: © 2024 by Walter de Gruyter Berlin/Boston
Copyright year: 2024
Copyright holder: De Gruyter
License: This work is licensed under the Creative Commons Attribution 4.0 International License.
License URL: https://creativecommons.org/licenses/by/4.0/

Page count: 5

==============================

Abstracts – DCK 2024 – 141st Congress of the German Society of Surgery – Leipzig, April 24–26 • DOI 10.1515/iss-2024-2004
Innov Surg Sci 2024; 9 (Special Suppl 1): s204–s208

**Nutcracker-Syndome (NCS) – therapy with transposition and anastomosis of superior mesenteric artery (SMA) onto the infrarenal aortic segment**
ID: 577

Raoul Friebe[1], Frank Meyer[2], Zuhir Halloul[3]

[1]*Otto-von-Guericke University with University Hospital, Division of Vascular Surgery; Dept. of General, Abdominal, Vacular and Transplant Surgery, Magdeburg, Deutschland*, [2]*Otto-von-Guericke University with University Hospital, Dept. of General, Abdominal, Vacular and Transplant Surgery, Magdeburg, Deutschland*, [3]*Otto-von-Guericke University with University Hospital, Division of Vascular Surgery; Dept. of General, Abdominal, Vacular and Transplant Surgery, Magdeburg*

**Background:**
Pain in the left flank and groin region comprises a broad spectrum of differential diagnosis.
Aim: To illustrate the rare diagnosis as well as a possible and suitable treatment option of NCS

**Materials and methods:**
Scientific case report based on **i)** clinical experiences obtained in the clinical and perioperative management of this single patient and **ii)** selective references from the medical scientific literature

**Results:**
(Case presentation): *Medical history and clinical finding:* A 32-years old male patient was admitted to the Dept. of Radiology for embolization of the left spermatic vein due to a testicular varicocele and pain in the groin region.
*Diagnostic measures:* Digital subtraction angiography (DSA) scans revealed a reversed flow in the left spermatic vein and pronounced collateral circulation of the left renal vein (LRV). A computed tomography(CT)-Angiography scan showed an angle between the longitudinal axis of superior mesenteric artery(SMA) and that of aorta of lower than 17°. Therefore, the finding was interpreted as the diagnosis “NCS”.
*Therapy:* The decision was made for a surgical treatment and the patient was electively admitted to the hospital. The surgical therapy comprised transposition of the SMA to the infrarenal section of the aorta.
*Postoperative course:* The patient was transferred to the ICU for one day of observation. On the following day, the patient was transferred back to the normal vascularsurgical ward with stable cardiopulmonary/-circulatory conditions. An immediate post-operative CT-Angio control showed no bleeding complication and regular visceral perfusion. Clinical status improved substantially, there were no further pain attacks.

**Conclusion:**
NCS is considered a rare diagnosis, with almost unknown prevalence. This diagnosis needs to be taken into account in case of left-side flank and groin pain especially in combination with proteinuria. A possible therapy of the NCS is the placement of a stent in the LRV. Due to the lack of data on the long-term-outcome in case of stent-implantation in the LRV, this approach remains for unoperable of high surgical risk subjects. Transposition of the SMA can be a good alternative therapeutic approach, with a more long-term prospect and prognosis, as shown in the presented case.

**References:**
1. Kurklinsky AK, Rooke TW. **Nutcracker Phenomenon and Nutcracker Syndrome.** *Mayo Clin Proc* 2010;85(6):552-9

2. Wang L, Yi L, Yang L, Liu Z, Rao J, Liu L, Yang J. **Diagnosis and Surgical Treatment of Nutcracker Syndrome: A Single-Center Experience.** *Urology* 2009;73(4):871-6

Open Access. © 2024 The Author(s), published by De Gruyter. This work is licensed under the Creative Commons Attribution 4.0 International License.

**Conformable stentgraft provides better reintervention free survival in blunt thoracic aortic injury**
ID: 702

Thomas Betz[1], Leszek Kukulski[2], Karin Pfister[1], Wilma Schierling[1], Georgios Sachsamanis[1], Ali Badrouni[1]

[1]*Uniklinik Regensburg, Franz-Josef-Strauss-Allee 11, Regensburg, Deutschland*, [2]*MEDICAL UNIVERSITY OF SILESIA, 18 Medyków Street, Katowice, Poland*

**Background:**
To analyze the impact the impact of anatomical and endograft factors on the outcome of Thoracic Endovascular Aortic Repair (TEVAR) for treatment of Blunt Thoracic Aortic Injury (BTAI).

**Materials and methods:**
A retrospective analysis of all patients with BTAI treated by TEVAR between June 2001 and May 2019 at a tertiary vascular and trauma center (Department of Vascular Surgery, University Medical Center Regensburg) was performed. Clinical data, procedural details, and anatomical and device related factors were recorded.

**Results:**
Overall 57 patients were analyzed (50 males; mean age 37 years). The most common trauma type was Grade III and IV. Thirty-day mortality was 3.5%, in-hospital mortality was 5,3%, respectively. In 30 cases a conformable device was used (cTAG, ©Gore). During a mean follow up of 4,3 years aortic related reintervention occurred in 14%. Aortic related reintervention was 25.9% in patients with non-conformable stent grafts and 3.3% in patients with conformable stent grafts (p=0,0142), respectively. Bird beak sign was observed in 61,4% of patients. Aortic related reintervention was 9.5% in patients with no bird peak sign after TEVAR and 16.7% in patients with bird peak sign after TEVAR (p=0,453), respectively.

**Conclusion:**
Aortic related reintervention is common after TEVAR in patients with BTAI. However, in our study conformable endografts showed a lower rate of aortic related reinterventions. These data should be taken into consideration for device selection for TEVAR in patients with BTAI.

**The Benefits of Non-Thermal, Non-Tumescent Cyanoacrylate Closure (VenasealTM) for Truncal Venous Insufficiency – Enough Evidence to Take over from Conventional Treatment Modalities?**
ID: 858

Richard Brandl[1]

[1]*Praxis für Gefäßchirurgie und Venenmedizin, München, Deutschland*

**Background:**
According to international guidelines, treatment of truncal insufficiency is widely dominated by thermal endovenous techniques, unless inherent risks and limitations in the face of lymphatic, nerval and thromboembolic complications. In recent years, cyanoacrylate closure (CC) initiated the era of a non-thermal/non-tumescent/non-sclerosant approach. Metaanalyses comparing CC with Laser (EVLA) or radiofrequency ablation (RFA) comprising a number of comparative studies, RCTs and observational studies show that in terms of effectiveness there is no significant difference between thermal and non-thermal modalities. In terms of safety, however, CC devices caused fewer adverse events and less severity at 12 months of follow-up than did EVLA or RFA.

**Materials and methods:**
Ten-year-experience with the VenaSeal™ closure system and review of the literature.

**Results:**
Advantages of CC are attributed to better patient comfort along with the intervention. CC devices caused less pain and shorter recovery times compared with RFA or EVLA. Furthermore, tumescent anesthesia and compression bandages are dispensable. Our ten-year-experience with the VenaSeal™ closure system confirms data in the literature and suggests substantially less long-term recurrencies due to neoangiogenesis compared with conventional surgery.

**Conclusion:**
Pathophysiological preconditions of specific side effects observed among different treatment options, implications for decision making and the need for generating further evidence are discussed.

Abstracts – DCK 2024 – 141st Congress of the German Society of Surgery – Leipzig, April 24–26 • DOI 10.1515/iss-2024-2004
Innov Surg Sci 2024; 9 (Special Suppl 1): s204–s208

**A Cost-Benefit Analysis of Endoscopic versus Conventional Vein Harvesting in Cardiac Surgery Based on the German DRG System**
ID: 38

Zulfugar Taghiyev[1], Markus Schloemicher[2], Yeong-Hoon Choi[3], Justus Strauch[2]

[1]*UKGM Giessen, Department of Cardiovascular Surgery, Gießen, Deutschland*, [2]*BG Universitätsklinikum Bergmannsheil Bochum, Department of Cardiothoracic Surgery, Bochum, Deutschland*, [3]*Kerckhoff-Klinik, Department of Cardiothoracic Surgery, Bad Nauheim, Deutschland*

**Background:**
A cost-benefit analysis of open vein harvesting (OVH) versus endoscopic vein harvesting (EVH) for leg wound complications in intermediate- and high-risk cardiac surgical patients was performed based on the German system of Diagnosis-Related Groups (G-DRG) in a retrospective cohort from 2018.

**Materials and methods:**
The highest Fowler score and EuroSCORE II were utilized as risk variables for leg wound infection. Risk adjustment (1:1) was performed to compare two groups of patients undergoing surgery with OVH or EVH techniques. Total costs, including costs of facilities, additional hospital stay, and personnel expenses based on Institute for the Hospital Remuneration System (InEK) calculations, were compared with G-DRG reimbursements.

**Results:**
The baseline characteristics of the two groups did not differ significantly. During the hospital stay, 3 (17.6%) patients in the OVH group had major leg-wound healing disorders. Patients in the OVH group were in the hospital slightly longer, although this was not statistically significant (14.3 vs 11.7 days; p=0.424). The total cost was 23,223€ for the OVH group compared with 18,627€ for the EVH group (p=0.000); thus, the cost of the EVH group exceeded that of the EVH group by 4,596€ based on G-DRG calculations (incl. endoscopic vessel harvesting systems).

**Conclusion:**
EVH was associated with significant cost savings and fewer leg wound complications in a group of patients with intermediate or high risk. The G-DRG reimbursement system ended with the statement that case-based flat rates are not viable for hospitals treating vulnerable groups of patients.

### Amnion Transplantation: A Promising Therapeutic Strategy for Wound Healing in Patients with Graft-Versus-Host Disease

ID: 818

Andreas Siegmund[1], Lukas Prantl[1], Sebastian Geis[1]

[1]*Abteilung für Plastische, Hand- und Wiederherstellungschirurgie, Abteilung für Plastische, Hand- und Wiederherstellungschirurgie, Regensburg, Deutschland*

**Background:**
Graft-versus-host disease (GVHD) is a significant complication of allogeneic hematopoietic stem cell transplantation, often resulting in severe and debilitating wounds. The only real alternative in case of failure of conservative therapy is the invasive transplantation of skin from the stem cell donor. This work presents the potential of human amniotic membrane (hAM) transplantation as a novel therapeutic approach for wound healing in a patient suffering from GVHD. The amniotic membrane, derived from the placenta, possesses unique properties including anti-inflammatory, anti-microbial, and anti-fibrotic effects, which are conducive to wound repair and regeneration.

**Materials and methods:**
Amniotic membrane transplantation was performed in three patients suffering from dermal GvHD disease. Human amniotic membrane was transplanted onto the soft tissue defect. We regularly monitored wound size and condition, incidence of infection, extent of local pain, and number of dressing changes.

**Results:**
In our three cases, complete wound closure was achieved in this patient within a few weeks with a single application of hAM. In addition, there was a local absence of pain and infection over the entire period, as well as no need for bandages after a few weeks.

**Conclusion:**
By means of transplantation of an amniotic membrane, complete healing of wounds resulting from GvHD reaction can be achieved. It thus offers an alternative to skin transplantation from the stem cell donor. Amnion transplantation as a potential game-changer in the management of GVHD-associated wounds paves the way for future research and clinical applications.
